# Matching-adjusted indirect comparison analysis of omalizumab versus dupilumab in patients with chronic spontaneous urticaria

**DOI:** 10.1016/j.jacig.2026.100668

**Published:** 2026-02-14

**Authors:** Giselle Mosnaim, Arpamas Seetasith, Michael Holden, Benjamin L. Trzaskoma, Sarbjit S. Saini

**Affiliations:** a Department of Medicine, Endeavor Health, Glenview, Ill; b Genentech Inc, South San Francisco, Calif; c Allergy and Immunology, Johns Hopkins Medicine, Baltimore, Md

**Keywords:** Chronic spontaneous urticaria, dupilumab, immunoglobulin E, matching-adjusted indirect comparison, omalizumab

## Abstract

**Background:**

Omalizumab is a well-established treatment for patients with chronic spontaneous urticaria, and dupilumab is a newly approved treatment. However, the lack of head-to-head randomized trials makes it challenging to compare treatment outcomes between omalizumab and dupilumab.

**Objective:**

To overcome this limitation, we used matching-adjusted indirect comparison to assess the efficacy of omalizumab versus dupilumab in primary phase 3 trials.

**Methods:**

Pooled patient-level data from the omalizumab ASTERIA I/II trials (NCT01287117/01292473) were compared with published aggregate data from the dupilumab LIBERTY-CSU CUPID Study A trial (NCT04180488). Populations were matched using the distribution of baseline weekly urticaria activity score (UAS7) in LIBERTY. Efficacy outcomes (UAS7; itch severity score [ISS7]) were then recalculated for the ASTERIA I/II trials using the matched cohorts. Least squares mean differences between treatments were determined by 2-sample *t* test. ClinicalTrials.gov identifiers: NCT01287117, 01292473, 04180488.

**Results:**

Improvements in both disease activity and itch severity were statistically significantly greater for omalizumab versus dupilumab (least squares mean [95% confidence interval] difference: UAS7, −7.6 [−13.0, −2.2], *P* = .006; ISS7, −3.0 [−5.8, −0.3], *P* = .031). Change from baseline in UAS7 at week 12 for omalizumab versus placebo was −12.6 (−15.7, −9.5) and for dupilumab versus placebo was −5.0 (−9.3, −0.7). Change from baseline in ISS7 at week 12 for omalizumab versus placebo was −5.4 (−6.9, −4.0) and for dupilumab versus placebo was −2.4 (−4.6, −0.1)

**Conclusions:**

Despite limitations, our matching-adjusted indirect comparison analysis highlights that omalizumab is an effective treatment option for appropriate patients with chronic spontaneous urticaria.

## Introduction

Omalizumab, an anti-IgE monoclonal antibody, is a well-established and approved treatment for patients with H_1_ antihistamine–refractory chronic spontaneous urticaria (CSU), with international guidelines recommending second-line omalizumab 300 mg every 4 weeks.^1^ Newer treatments include dupilumab, a monoclonal antibody that inhibits IL-4 and IL-13 signaling,^2^ which has recently been approved in the United States for patients with CSU. However, the lack of head-to-head randomized trials makes it challenging to compare treatment outcomes between omalizumab and dupilumab. To overcome this limitation, matching-adjusted indirect comparison (MAIC) analyses can account for cross-trial differences and allow for reliable indirect comparisons to be made across separate trials, for which patient-level data are available for one treatment and published aggregate data are available for a comparator treatment.^3^ MAIC is thus applicable to comparing omalizumab (patient-level data) versus dupilumab (published aggregate data) for CSU. Importantly, given the rapidly evolving treatment landscape for CSU, MAIC analyses may aid physicians in making informed treatment decisions when assessing their patients with CSU. Therefore, in this study, we used a published MAIC methodology^4^ to assess the efficacy of omalizumab (patient-level data) versus dupilumab (published aggregate data) in primary phase 3 trials.

In this MAIC analysis, pooled data from the omalizumab ASTERIA I/II trials (NCT01287117, 01292473), which included patients aged ≥12 years with CSU who continued to receive a standard dose of H_1_ antihistamines,^5^^,^^6^ were compared with published aggregate data from the dupilumab LIBERTY-CSU CUPID Study A trial (NCT04180488), which included omalizumab-naïve patients aged ≥6 years with CSU who continued to receive their background dose of H_1_ antihistamines (up to 4 times the standard dose).^2^ A comparison of study designs, including dosing schedules and primary end points, is presented in Fig E1 in the Online Repository available at www.jaci-global.org. Using published MAIC methodology from Malagone and Sherman^4^ (further detailed in Jiang et al,^7^ and an example is provided in Bourdin et al^8^), individual patient data from ASTERIA I/II were matched to available summary data from LIBERTY-CSU CUPID Study A using the distribution of baseline weekly urticaria activity score (UAS7) as the matching variable. (In LIBERTY-CSU CUPID Study A, 29.7% of patients had UAS7 < 28 and 70.3% of patients had UAS7 ≥ 28.) Random samples from ASTERIA I/II (N = 1000) were drawn without replacement to match the comparator distributions within treatment arms (see Table E1 in the Online Repository). Efficacy outcomes were then recalculated for the ASTERIA I/II trials using the matched cohorts. Change from baseline at week 12 in UAS7 and weekly itch severity score (ISS7) for placebo, omalizumab 300 mg, and dupilumab 300 mg were determined; least squares mean (LSM) differences with 95% confidence intervals (CIs) are shown. Differences between treatments (omalizumab vs placebo, dupilumab vs placebo) were determined by 2-sample *t* tests. Analyses were verified by sensitivity analyses assessing other matching variables (no differences were observed) and by independent validation (by EVEREST Clinical Research; everestclinical.com).

## Results and discussion

**Fig 1:**
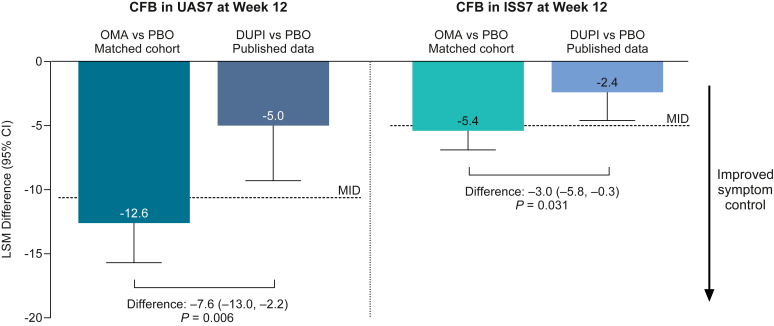
CFB in UAS7 and ISS7 at week 12. LSM difference (95% CI) between placebo and treatment. Published MIDs: ISS7, −4.5, −5.0; UAS7, −9.5, −10.5.^7^*CFB,* Change from baseline; *DUPI,* dupilumab; *MID,* minimal important difference; *OMA,* omalizumab; *PBO,* placebo.

**Fig 2:**
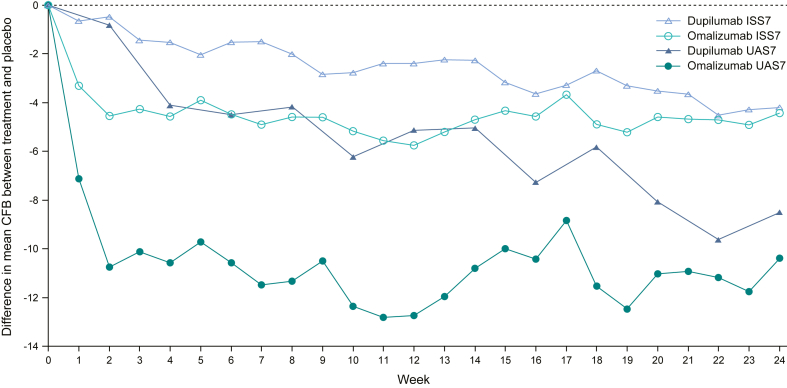
Mean CFB difference in UAS7 and ISS7 up to week 24 between placebo-adjusted omalizumab and placebo-adjusted dupilumab. For placebo-adjusted dupilumab, published data points were used where possible. For all other data points, tracing software was used from published figures. *CFB,* Change from baseline.

Improvements in both disease activity and itch severity were statistically significantly greater for omalizumab versus dupilumab (Fig 1; LSM [95% CI] difference: UAS7, −7.6 [−13.0, −2.2], *P* = .006; ISS7, −3.0 [−5.8, −0.3], *P* = .031). For disease activity, change from baseline in UAS7 at week 12 for omalizumab versus placebo was −12.6 (−15.7, −9.5) and for dupilumab versus placebo was −5.0 (−9.3, −0.7). The minimal important difference in UAS7 was reached with omalizumab treatment only.^9^ For itch severity, LSM (95% CI) change from baseline in ISS7 at week 12 for omalizumab versus placebo was −5.4 (−6.9, −4.0) and for dupilumab versus placebo was −2.4 (−4.6, −0.1) (Fig 1). The minimal important difference in ISS7 was reached with omalizumab treatment only.^9^ Mean change from baseline over time (to week 24) for UAS7 and ISS7 for placebo-adjusted omalizumab and dupilumab is shown in Fig 2; differences between omalizumab and dupilumab are less at week 24 versus week 12. Overall, improvements in UAS7 and ISS7 at week 12 were statistically significantly greater for omalizumab versus dupilumab, suggesting faster symptom control with omalizumab, and this pattern was evident from week 1.

By MAIC analysis, omalizumab demonstrated statistically superior week 12 efficacy versus dupilumab in patients with CSU. Because MAIC is an established and published methodology,^4^ our findings allow for a comparison of efficacy outcomes in the absence of head-to-head clinical trials. Our findings are in line with a recent meta-analysis that found improved efficacy with omalizumab 300 mg versus dupilumab.^10^ Both biologics are generally well tolerated across CSU primary phase 3 trials^2^^,^^5^^,^^6^ and across indications.^11^

There are some limitations to our analysis because several cross-trial differences exist between ASTERIA I/II and LIBERTY-CSU CUPID Study A, including patient demographics and clinical characteristics, different background treatments (standard H_1_ antihistamines for ASTERIA I/II vs up to 4 times standard H_1_ antihistamines for LIBERTY-CSU CUPID Study A), years during which the trials were conducted (ASTERIA I/II trials were conducted during 2011-12; LIBERTY-CSU CUPID Study A was conducted during 2019-24), and time when primary end point was measured (week 12 for ASTERIA I/II and week 24 for LIBERTY-CSU CUPID Study A). These cross-trial differences, especially baseline antihistamine receipt (due to the timing of the trials), could have influenced the disease severity of patients entering each study and thus our findings may be affected by selection bias; however, mean itch severity, hives severity, disease activity, quality of life, and angioedema were all similar in patients enrolled onto ASTERIA I/II and LIBERTY-CSU CUPID Study A.^2^^,^^5^^,^^6^

In conclusion, despite limitations, our MAIC analysis highlights that omalizumab is an effective treatment option for appropriate CSU patients. Omalizumab showed statistically greater symptom improvement at week 12 compared with dupilumab, with differences observed as early as 1 week after treatment; this may result in concurrent improvements in patient quality of life. Although a direct head-to-head trial is needed for confirmation, our findings may help physicians and patients make informed treatment decisions for this debilitating chronic disease.

**Clinical implication**

This MAIC of primary phase 3 omalizumab and dupilumab trials demonstrates statistically significantly greater placebo-adjusted improvements in disease activity and itch severity at week 12 for omalizumab versus dupilumab in patients with CSU.

## Disclosure statement

The ASTERIA I/II trials were funded by 10.13039/100004328Genentech, a member of the Roche Group, and Novartis Pharma AG. The LIBERTY-CSU CUPID Study A was funded by Sanofi-Regeneron, and only published data were used. Genentech Inc was involved in the study design, data analysis, and preparation of the report. Medical writing assistance was provided by Janelle Keys, PhD, CMPP, and Nilisha Fernando, PhD, of Envision Pharma Group, and was funded by Genentech. Envision Pharma Group’s services complied with international guidelines for Good Publication Practice (GPP 2022).

Data sharing statement: For eligible studies, qualified researchers may request access to individual patient-level clinical data through a data request platform, available at Vivli (vivli.org/ourmember/roche/). Up-to-date details on Roche’s Global Policy on the Sharing of Clinical Information and how to request access to related clinical study documents are available online (go.roche.com/data_sharing). Anonymized records for individual patients across more than one data source external to Roche cannot, and should not, be linked because of a potential increase in risk of patient reidentification.

Disclosure of potential conflict of interest: G. Mosnaim receives current research grant support from Areteia, Celldex, GlaxoSmithKline, Genentech, Incyte, Merck, Novartis, Sanofi-Regeneron, and Teva; and receives consulting, advisory board, and/or speaking fees from Abbott, Chiesi, Genentech, Jasper, Novartis, Sanofi-Regeneron, and Teva. A. Seetasith, M. Holden, and B. L. Trzaskoma are employees of Genentech Inc and stockholders in Roche. S. S. Saini has received research/grant/clinical trial support from Allakos, Amgen, Escient, the National Institutes of Health, Novartis, and Sanofi-Regeneron; and has been a consultant and/or advisory board member for Allakos, Aquestive, Celltrion, Escient, Granular Therapeutics, Innate Therapies, Novartis, and Sanofi-Regeneron.

## Supplementary data

Supplementary Fig E1

Supplementary Table E1
