# Supplementary material for: Matching-adjusted indirect comparison analysis of omalizumab versus dupilumab in patients with chronic spontaneous urticaria
Source: J Allergy Clin Immunol Glob. 2026 Feb 14;5(3):100668. doi: 10.1016/j.jacig.2026.100668 (PMC12990347; doi:10.1016/j.jacig.2026.100668)
Supplement: Supplementary Fig E1 [file mmc1.pdf]

FIGURE E1. Comparison of ASTERIA I/II (omalizumab)[E1,E2] and LIBERTY-CSU CUPID Study A (dupilumab)[E3] study designs. *In addition, all patients received an H1-antihistamine. *CFB*, change from baseline; *ISS7*, weekly itch severity score; *Q2W*, every 2 weeks; *Q4W*, every 4 weeks; *UAS7*, weekly urticaria activity score.

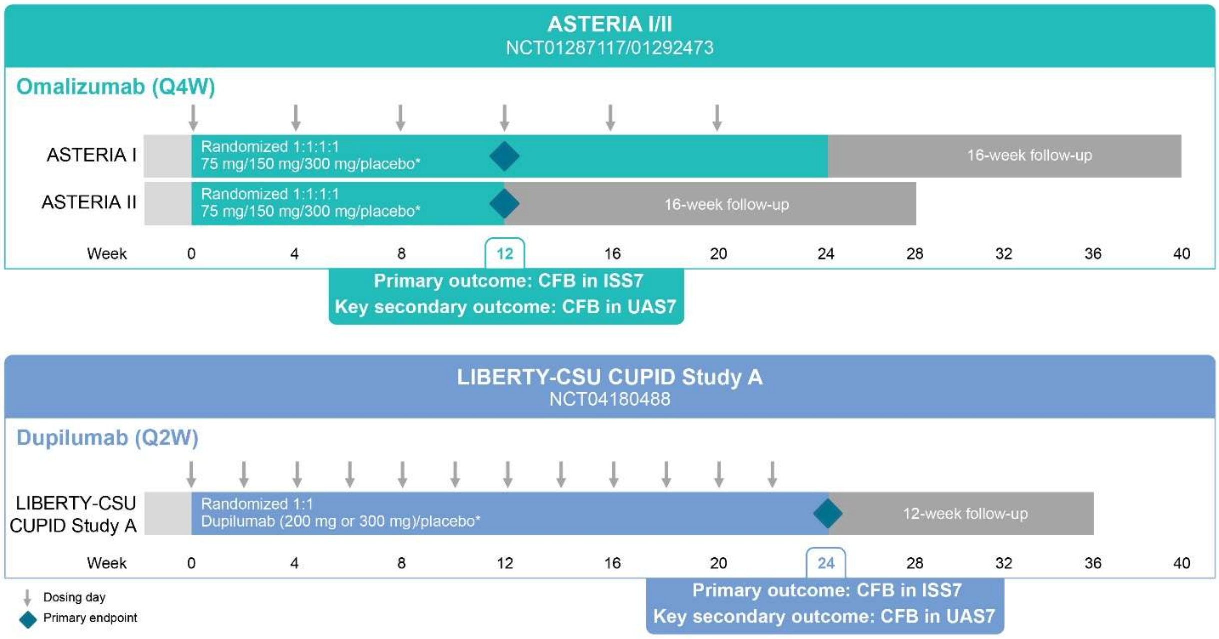

Online Repository References

E1. Saini SS, Bindslev-Jensen C, Maurer M, Grob J-J, Bülbül Baskan E, Bradley MS, et al. Efficacy and safety of omalizumab in patients with chronic idiopathic/spontaneous urticaria who remain symptomatic on H_1 antihistamines: a randomized, placebo-controlled study. J Invest Dermatol 2015;135:67-75.

E2. Maurer M, Rosén K, Hsieh H-J, Saini S, Grattan C, Gimenéz-Arnau A, et al. Omalizumab for the treatment of chronic idiopathic or spontaneous urticaria. N Engl J Med 2013;368:924-35.

E3. Maurer M, Casale TB, Saini SS, Ben-Shoshan M, Giménez-Arnau AM, Bernstein JA, et al. Dupilumab in patients with chronic spontaneous urticaria (LIBERTY-CSU CUPID): two randomized, double-blind, placebo-controlled, phase 3 trials. J Allergy Clin Immunol 2024;154:184-94.
