# Supplementary material for: Matching-adjusted indirect comparison analysis of omalizumab versus dupilumab in patients with chronic spontaneous urticaria
Source: J Allergy Clin Immunol Glob. 2026 Feb 14;5(3):100668. doi: 10.1016/j.jacig.2026.100668 (PMC12990347; doi:10.1016/j.jacig.2026.100668)
Supplement: Supplementary Table E1 [file mmc2.pdf]

TABLE E1. Matching comparator distributions*

	Placebo		Treatment	
	Baseline UAS7 <28	Baseline UAS7 ≥28	Baseline UAS7 <28	Baseline UAS7 ≥28
Target distribution from LIBERTY-CSU CUPID A, %	35.3	64.7	24.3	75.7
Observed distribution from ASTERIA I/II, n (%)	45 (28.3)	114 (71.7)	55 (34.4)	105 (65.6)
Matched distribution from ASTERIA I/II, n (%)	44 (35.2)	81 (64.8)	33 (24.3)	103 (75.7)

*1000 random samples were selected without replacement, utilizing the sample sizes determined per treatment to match the comparator distributions within treatment. The matching variable utilized for the analysis was disease severity (baseline UAS7).

UAS7, weekly urticaria activity score.
